# Genome-Wide Comparative Analysis of the *R2R3-MYB* Gene Family in Six *Ipomoea* Species and the Identification of Anthocyanin-Related Members in Sweet Potatoes

**DOI:** 10.3390/plants12081731

**Published:** 2023-04-21

**Authors:** Maoxing Li, Yuanping Zhou, Kaifeng Li, Huachun Guo

**Affiliations:** 1 College of Agronomy and Biotechnology, Yunnan Agricultural University, Kunming 650201, China; limaoxing2011@163.com (M.L.); yuanping-zhou@outlook.com (Y.Z.); dtllx01@sina.cn (K.L.); 2 Yunnan Engineering Research Center of Tuber and Root Crop Bio-Breeding and Healthy Seed Propagation, Yunnan Agricultural University, Kunming 650201, China

**Keywords:** *Ipomoea* species, sweet potato, *R2R3-MYB* gene family, expression analysis, anthocyanin biosynthesis

## Abstract

Sweet potatoes (*Ipomoea batatas*) are one of the important tuberous root crops cultivated worldwide, and thier storage roots are rich in antioxidants, such as anthocyanins. *R2R3-MYB* is a large gene family involved in various biological processes, including anthocyanin biosynthesis. However, few reports about the *R2R3-MYB* gene family of sweet potatoes have been released to date. In the present study, a total of 695 typical *R2R3-MYB* genes were identified in six *Ipomoea* species, including 131 *R2R3-MYB* genes in sweet potatoes. A maximum likelihood phylogenetic analysis divided these genes into 36 clades, referring to the classification of 126 R2R3-MYB proteins of Arabidopsis. Clade C25(S12) has no members in six *Ipomoea* species, whereas four clades (i.e., clade C21, C26, C30, and C36), including 102 members, had no members in Arabidopsis, and they were identified as *Ipomoea*-specific clades. The identified *R2R3-MYB* genes were unevenly distributed on all chromosomes in six *Ipomoea* species genomes, and the collinearity analysis among hexaploid *I. batatas* and another five diploid *Ipomoea* species suggested that the sweet potato genome might have undergone a larger chromosome rearrangement during the evolution process. Further analyses of gene duplication events showed that whole-genome duplication, transposed duplication, and dispersed duplication events were the primary forces driving the *R2R3-MYB* gene family expansion of *Ipomoea* plants, and these duplicated genes experienced strong purifying selection because of their Ka/Ks ratio, which is less than 1. Additionally, the genomic sequence length of 131 *IbR2R3-MYBs* varied from 923 bp to ~12.9 kb with a mean of ~2.6 kb, and most of them had more than three exons. The Motif 1, 2, 3, and 4 formed typical R2 and R3 domains and were identified in all IbR2R3-MYB proteins. Finally, based on multiple RNA-seq datasets, two *IbR2R3-MYB* genes (*IbMYB1/g17138.t1* and *IbMYB113/g17108.t1*) were relatively highly expressed in pigmented leaves and tuberous root flesh and skin, respectively; thus, they were identified to regulate tissue-specific anthocyanin accumulation in sweet potato. This study provides a basis for the evolution and function of the *R2R3-MYB* gene family in sweet potatoes and five other *Ipomoea* species.

## 1. Introduction

Sweet potato (*Ipomoea batatas* (L.) Lam., 2*n* = 6x = 90), of the genus *Ipomoea* from the Convolvulaceae family, is an important tuberous root crop cultivated globally [1,2]. Currently, China is the largest sweet potato cultivation and production region; China accounts for approximately 54% of the world’s production and more than 29% of the global area [3]. Sweet potato storage roots are rich in carbohydrates, dietary fibers, minerals, vitamins, and various antioxidants, such as carotenoids and anthocyanins, which are beneficial to human health [4]. Anthocyanins, belonging to flavonoids metabolites, are natural water-soluble pigments accumulated in vacuoles that are responsible for the red, purple, and blue coloration of plant tissues [5]. Anthocyanins are distributed among flowers, leaves, stems, and storage roots of sweet potatoes, such as the storage roots of purple-fleshed sweet potatoes, which are rich in anthocyanins [6,7,8]. Sweet potato tissue coloration involves complex biochemical changes due to genetic and environmental factors, and anthocyanin accumulation in different tissues exhibits no correlation. Hence, understanding the genetic factors controlling anthocyanin biosynthesis and accumulation is valuable for sweet potato breeding and germplasm resource utilization.

V-myb avian myeloblastosis viral oncogene homolog (MYB) proteins are the second largest transcription factor (TF) families in higher plants [9,10]. MYB TFs are composed of a highly conserved DNA-binding domain repeat (R) at the N-terminal and a relatively variable regulatory region at the C-terminal. Each MYB repeat (R) harbors approximately 52 amino acid residues, among which 3 regularly spaced tryptophan (W) residues form 3 α-helices [11]. Typically, according to the MYB repeats sequence similarity and repeat number, plant MYB proteins contain one to four imperfect MYB repeat(s) and are, therefore, classified as 1R-MYBs (R3-MYBs and MYB-related), 2R-MYBs (R2R3-MYBs), 3R-MYBs (R1R2R3-MYBs), and 4R-MYBs (R1R2R2R1/2-MYBs) [9,12,13]. The R2R3-MYB gene family members have expanded substantially during plant evolution and constitute the predominant family [10], which contributes to R2R3-MYB becoming an important research focus.

Plant *R2R3-MYB* genes perform crucial roles in various biological processes, including plant growth and development, cell differentiation, specialized secondary metabolism, and biotic and abiotic stress responses [14,15]. In particular, many *R2R3-MYB* genes have been thoroughly investigated and verified to regulate tissue-specific anthocyanin accumulation in various plants [16,17,18]. For example, the first *R3R3-MYB* plant gene, *COLORED1* (*C1*), which was isolated from maize, encodes a regulatory protein to control anthocyanin pigment production in the aleurone layer of the endosperm [19,20]. In Arabidopsis, the overexpression of *AtMYB75/PAP1* or *AtMYB90/PAP2* produces enhanced anthocyanin pigmentation throughout the entire plant [21]. In addition, the overexpression of *AtMYB113* or *AtMYB114* results in substantial increases in pigment production in vegetative tissues [22]. In tomatoes, the dominant *Anthocyanin fruit* (*Aft*) locus encodes the R2R3-MYB TF named SlAN2-like, which acts as a functional anthocyanin activator that is responsible for light-dependent anthocyanin accumulation in the fruit skin of the purple tomato variety ‘Indigo Rose’ [23]. In apples, three R2R3-MYB TFs, namely, MdMYB1, MdMYBA, and MdMYB10, are the key regulators in apple fruit anthocyanin accumulation and coloration [24,25,26]. In carrots, two *R2R3-MYB* genes, namely, *DcMYB7* and *DcMYB113*, perform important roles in regulating anthocyanin biosynthesis, of which, *DcMYB7* has a high expression level in carrot root and petiole [27], but *DcMYB113* is specifically expressed in carrot root [28]. In addition, some R2R3-MYB repressors were also reported, such as PhMYB27 in petunias [29], FaMYB1 in strawberries [30], and StMYB44 in potatoes [31]. These studies summarized that R2R3-MYB TFs are the essential regulators for anthocyanin biosynthesis in plants and that S6 (subgroup 6) R2R3-MYB TFs are conserved as anthocyanin activators in many plants referencing the Arabidopsis *R2R3-MYB* gene family [9].

The anthocyanin biosynthetic pathway is highly conserved and has been well-studied in many plants [22,32]. Several key enzyme-coding genes, including *IbCHS*, *IbCHI*, *IbF3’H*, *IbDFR*, *IbANS*, *Ib3GGT*, and *IbGSTF4,* have been linked to anthocyanin biosynthesis and accumulation within sweet potatoes [33,34,35,36,37,38]. An *R2R3-MYB* gene, *IbMYB1-2*, which is specifically highly expressed in purple-fleshed sweet potato storage root flesh, is considered the main factor for activating the anthocyanin biosynthesis and accumulation in tuberous roots [6,39,40,41]. IbMYB44, the R2R3-MYB repressor, competitively inhibits the IbMYB340-IbbHLH2-IbNAC56a/b complex’s formation to affect anthocyanin biosynthesis in purple-fleshed sweet potatoes [42]. Moreover, it was reported that multiple MYB TFs (i.e., activators IbMYB1/2/3, repressors IbMYB27, IbMYBx, and IbMYB4a/b/c) co-regulate the accumulation of anthocyanins in sweet potato leaves [43]. Despite preliminary exploration, the underlying transcriptional regulatory mechanism of *R2R3-MYBs* in the anthocyanin metabolism pathway has not been systematically investigated in sweet potatoes.

In recent decades, the increasing availability of plant genome sequencing data has made comprehensively characterizing the genome-wide *R2R3-MYB* gene family in the plant kingdom successful. Currently, this gene family has been identified and characterized in more than 75 plant species [13,14], including Arabidopsis (*Arabidopsis thaliana*) [9], rice (*Oryza sativa*) [44], potato (*Solanum tuberosum*) [45], tomato (*Solanum lycopersicum*) [46], wolfberry (*Lycium barbarum*) [47], and pea (*Pisum sativum*) [48]. Nevertheless, little is known about the information of the genome-wide *R2R3-MYB* gene family in sweet potatoes. Moreover, there are no reports about the comparative analysis of *R2R3-MYB* genes in the genus *Ipomoea*, which includes approximately 500 species and possesses the largest number of species in the Convolvulaceae family [49]. In recent years, the chromosome-level genomes of six *Ipomoea* species, including the hexaploid species *Ipomoea batatas*, and five other diploid species, including *Ipomoea trifida*, *Ipomoea triloba*, *Ipomoea nil*, *Ipomoea purpurea*, and *Ipomoea aquatica,* have been sequenced, assembled, annotated, and released [50,51,52,53,54]. The genome information on these wild diploid relatives has been used in genome-enabled breeding and the research of sweet potatoes. Therefore, these genomes sequence data are informative for a comparative analysis of the R2R3-MYB TF gene family among the *Ipomoea* species.

In this study, a genome-wide comparative analysis of the *R2R3-MYB* gene family was performed. First, a total of 695 *R2R3-MYB* genes were identified in 6 *Ipomoea* species. Next, the protein physicochemical properties, phylogenetic relationships, chromosome localization, syntenic links, gene duplication events, gene structures, and conserved motifs of identified *R2R3-MYB* genes were analyzed. The expression patterns of anthocyanin-related *IbR2R3-MYBs* in sweet potatoes with different colored tissues were also investigated based on public RNA-seq data. These findings will provide valuable information for further studies of the evolution and function of *R2R3-MYB* gene families in *Ipomoea* species.

## 2. Results

### 2.1. Identification and Characterization of R2R3-MYB Family Members in Six Ipomoea Species

**Figure 1:**
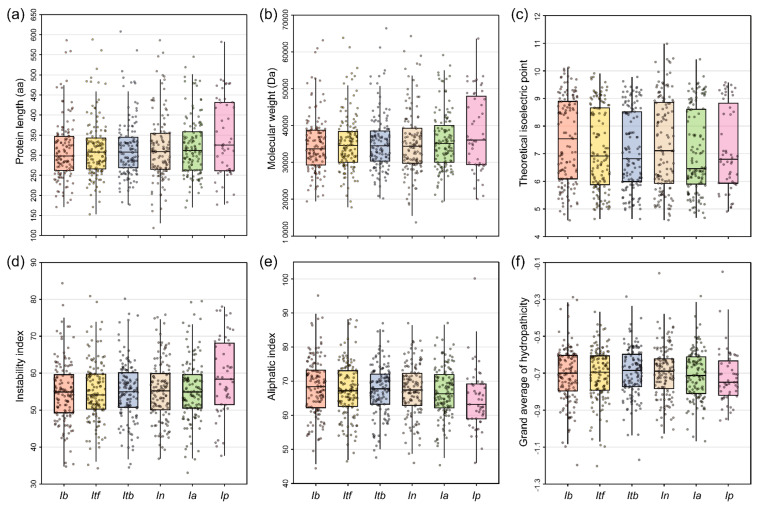
Physicochemical properties of all identified R2R3-MYB proteins in six *Ipomoea* species. (**a**) Protein length; (**b**) Molecular weight (MW); (**c**) Isoelectric point (pI); (**d**) Instability index; (**e**) Aliphatic index; (**f**) Grand average of hydropathicity (GRAVY).

Three strategies (i.e., BLASTP search, HMM search, and SMART database validation) were used to fully identify R2R3-MYB TFs in six *Ipomoea* species. A total of 695 typical R2R3-MYB TFs were identified, including 131 in sweet potato, 133 in *I. trifida*, 129 in *I. triloba*, 127 in *I. nil*, 124 in *I. aquatica*, and 51 in *I. purpurea* (Table 1). Next, to further characterize all R2R3-MYB proteins features, the physiochemical properties, including protein length, molecular weight (MW), isoelectric point (pI), instability index, aliphatic index, and grand average of hydropathicity (GRAVY), were analyzed (Figure 1) and the subcellular localization of proteins was also predicted (Table S1). These results showed that amino acid numbers in the 6 *Ipomoea* R2R3-MYB proteins ranged from 119 to 608, with an average of 318 amino acids (Figure 1a). The MW varied from 13.71 to 66.40 kDa, and itb04g32410.t2 had the longest length of 608 amino acids and the highest molecular weight of 66.40 kDa, while INIL06g37520.t1 had the shortest length of 119 amino acids and the lowest molecular weight of 13.71 kDa (Figure 1b). The pI ranged between 4.59 (g29760.t1) and 10.98 (INIL00g10723.t1), among which, 50.8% (353/695) of proteins are acidic proteins with a pI that is lower than 7, and the rest are basic proteins with a pI that is larger than 7 (Figure 1c). The instability index of 3.0% (221/695) of the proteins was less than 40, while the rest exhibited more than 40, suggesting that they are unstable proteins (Figure 1d). The aliphatic index was distributed from 44.45 (g48266.t1) to 100.16 (Ipurp_gene18717) (Figure 1e). The GRAVY of all proteins was less than −0.1, indicating that all R2R3-MYB proteins had negative hydrophobicity (Figure 1f). Moreover, the prediction of subcellular localization showed that all R2R3-MYB proteins were localized in the nucleus (Table S1), suggesting their critical role in regulatory functions.

### 2.2. Phylogenetic Analysis and Classification of R2R3-MYB Genes in Six Ipomoea Species

**Figure 2:**
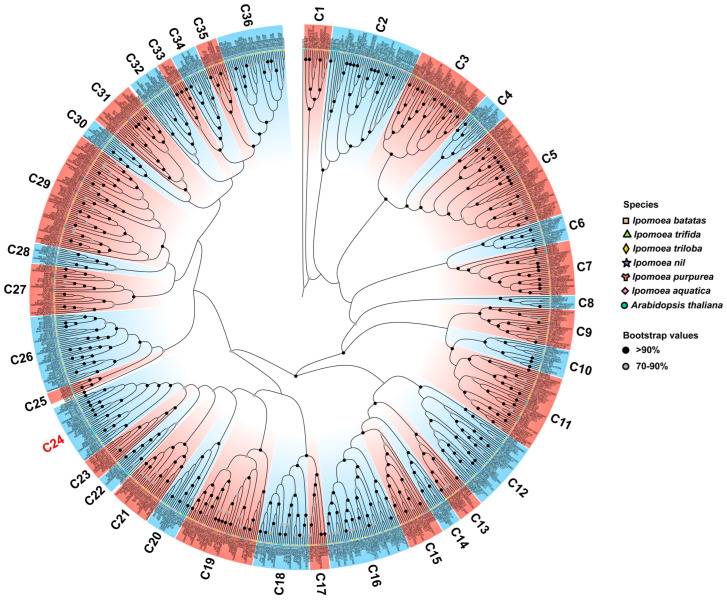
Phylogenetic tree of R2R3-MYB members in Arabidopsis and six *Ipomoea* species. All identified R2R3-MYB members from *I. batatas* (131), *I. trifida* (133), *I. triloba* (129), *I. nil* (127), *I. purpurea* (51), *I. aquatica* (124), and Arabidopsis (126) were used. The full-length amino acid sequences of R2R3-MYB proteins were aligned using MAFFT, and the tree was constructed by the maximum-likelihood (ML) method of IQ-tree using the JTT + F + R10 model. These R2R3-MYB proteins are clustered into 36 clades (designated as C1 to C36). The AtCDC5 was rooted as the outgroup, and 4 proteins did not fit well into the clade. The genes that belonged to the same organism were marked in the same shape and color. The dots on the branches represent bootstrap values based on 1000 replications, and bootstrap values >90% and ≥70% are shown as black and gray dots in the phylogenetic tree, respectively, while those <70% are not shown.

To investigate the evolutionary relationships and gene functions of *R2R3-MYB* genes in 6 *Ipomoea* species, a rooted maximum likelihood (ML) phylogenetic tree consisting of 126 members from Arabidopsis and 695 members from 6 *Ipomoea* was constructed via MAFFT software for multiple sequence alignment and IQ-tree software was used for phylogenetic tree clustering. According to the topology of the ML phylogenetic tree and referring to the classification of AtR2R3-MYB family proteins [9,14], all R2R3-MYB proteins were subdivided into 36 clades (named C1-C36) (Figure 2 and Table S2), except for 4 AtR2R3-MYB proteins (i.e., AtMYB123, AtMYB39, AtMYB47, and AtMYB95). Clade C25 had six members only in Arabidopsis and not in *Ipomoea* species, and they were Arabidopsis-specific clades. While clades C21, C26, C30, and C36 had no members in Arabidopsis, they were identified as *Ipomoea*-specific clades. However, there were no sweet potato-specific clades. Clade C29 had the highest number of *R2R3-MYB* genes, containing 9, 11, 10, 9, and 12 genes in sweet potatoes, *I. trifida*, *I. triloba*, *I. nil*, and *I. aquatica*, respectively. Meanwhile, some clades (i.e., C8, C22, and C30) had only one member for each *Ipomoea* species (Table 2). These results showed that the number of R2R3-MYB proteins among different clades was highly variable, suggesting *R2R3-MYB* gene diversity in *Ipomoea* species. Meanwhile, the number of R2R3-MYB members of sweet potatoes in each clade was almost similar with other *Ipomoea* species but different with Arabidopsis, such as C3–C6, suggesting *R2R3-MYB* gene conservation within the same clade of *Ipomoea* species.

Research progress related to *R2R3-MYB* gene function has shown that the members grouped in the same clade might exhibit a similar biological function, and these R2R3-MYB members are involved as activators or repressors in transcriptional regulations [14]. The C24(S6), C19(S4), C20(S7), and C23(S44) clades are known to participate in phenylpropanoid-derived flavonoids biosynthesis and the metabolism pathway, including the regulation of the anthocyanin’s biosynthesis pathway. For example, C24 clade members containing AtMYB75 (PAP1), ATMYB90 (PAP2), AtMYB113, and AtMYB114, were proven to positively regulate anthocyanin biosynthesis [21,22], indicating that these twenty-six R2R3-MYB TFs (i.e., 4, 6, 5, 6, and 5 in sweet potatoes, *I. trifida*, *I. triloba*, *I. nil*, and *I. aquatica*, respectively) may control anthocyanin biosynthesis and metabolism in *Ipomoea* plants.

### 2.3. Chromosomal Distribution and Synteny Analysis of R2R3-MYBs in Six Ipomoea Species

**Figure 3:**
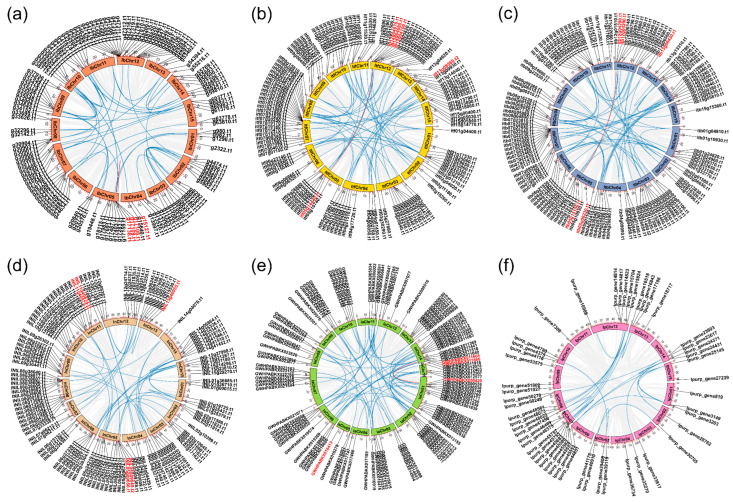
Chromosomal distribution and intraspecies synteny analysis of *R2R3-MYBs* in six *Ipomoea* species. (**a**) *I. batatas*; (**b**) *I. trifida*; (**c**) *I. triloba*; (**d**) *I. nil*; (**e**) *I. aquatica*; (**f**) *I. purpurea*. The circle represents 15 chromosomes of each *Ipomoea* species. The *R2R3-MYBs* are unevenly distributed on corresponding all chromosomes in six *Ipomoea* species’ genomes. The blue lines show syntenic gene pairs of *R2R3-MYBs*, and the red lines show syntenic gene pairs of *R2R3-MYBs* belonging to clade C24(S6). The detailed information is listed in Table S4.

Based on the corresponding chromosome-level genome sequence and annotation information, the 131, 128, 128, 123, 124, and 51 *R2R3-MYB* genes were mapped throughout the 15 chromosomes of *I. batatas*, *I. trifida*, *I. triloba*, *I. nil*, *I. aquatica*, and *I. purpurea*, respectively (Figure 3). The remaining genes (5 in *I. trifida*, 1 in *I. triloba*, 4 in *I. nil*, and 15 in *I. aquatica*) were located on unanchored scaffolds and have not been mapped (Table S3). Chromosome numbering was assigned and named “IbChr”, “ItfChr”, “ItbChr”, “InChr”, “IaChr”, and “IpChr”. The chromosomal location results showed that the *R2R3-MYB* genes were distributed on all chromosomes in six *Ipomoea* species’ genomes, but the distribution appeared to be uneven. For the *I. batatas* genome, IbChr12 had the largest number of *IbR2R3-MYB* genes (*n* = 24), while IbChr06 had the smallest number (*n* = 3). Among the other chromosomes, 14 *IbR2R3-MYBs* were detected in IbChr05, 12 in IbChr07, 11 in IbChr02, 10 in IbChr09, 9 in IbChr08, 8 in IbChr11, 7 in IbChr14, 6 in IbChr03, IbChr04, IbChr10 and IbChr15, 5 in IbChr13, and 4 in IbChr01. For *I. trifida* and *I. triloba* genomes, the distribution of *R2R3-MYBs* was highly similar, Itf/ItbChr07 had the largest number (*n* = 20/18), and Itf/ItbChr15 had the smallest number (*n* = 4/4). However, *R2R3-MYB* genes were randomly distributed in the *I. nil*, *I. aquatica*, and *I. purpurea* genomes. For example, the highest numbers of *R2R3-MYBs* were distributed inInChr08 (*n* = 15), IaChr01 and IaChr15 (*n* = 15), and IpChr07 (*n* = 9) in *I. nil*, *I. aquatica*, and *I. purpurea*, respectively. These results indicated that the distribution of *R2R3-MYBs* is different and disproportionate on chromosomes in sweet potatoes and five other *Ipomoea* species.

**Figure 4:**
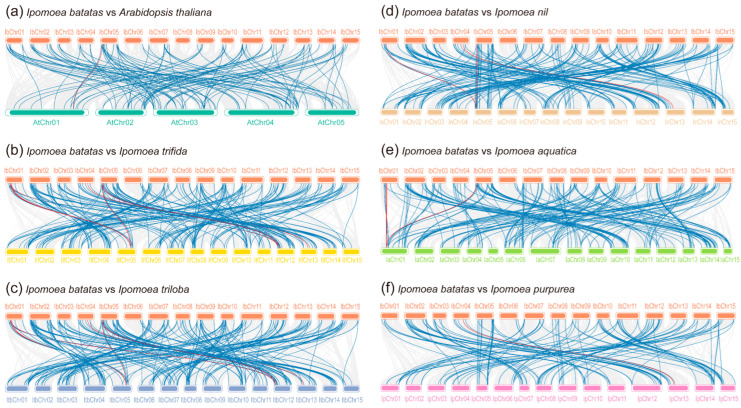
Interspecies collinear analysis of *R2R3-MYBs* between *I. batatas* and Arabidopsis, and other five *Ipomoea* species. (**a**) Collinear analysis of *IbR2R3-MYBs* between *AtR2R3-MYBs*. (**b**) Collinear analysis of *IbR2R3-MYBs* between *ItfR2R3-MYBs*. (**c**) Collinear analysis of *IbR2R3-MYBs* between *ItbR2R3-MYBs*. (**d**) Collinear analysis of *IbR2R3-MYBs* between *InR2R3-MYBs*. (**e**) Collinear analysis of *IbR2R3-MYBs* between *IaR2R3-MYBs*. (**f**) Collinear analysis of *IbR2R3-MYBs* between *IpR2R3-MYBs*. The gray line links show the collinear block with *I.batatas* and six other plant species’ genomes. The blue lines show collinear gene pairs related to *R2R3-MYBs*, while the red lines show collinear gene pairs related to *R2R3-MYBs* belonging to clade C24(S6). The detailed information is listed in Table S5.

This study identified further intraspecific synteny blocks for each species. The results showed that there were 301 syntenic gene pairs among the 6 *Ipomoea* species. Of these, sweet potato, *I. trifida*, *I. triloba*, *I. nil*, *I. aquatica*, and *I. purpurea* had 46, 60, 61, 41, 68, and 25 gene pairs, respectively (Figure 3 and Table S4). One syntenic gene pair on IbChr05 (*g17105.t1-g17138.t1*) in the sweet potato genome, the *itf05g14440.t1-itf12g04070.t1* pair in *I. trifida*, the *itb05g15100.t1-itb12g04280.t1* pair in *I. triloba*, and the *GWHPABKX000429-GWHPABKX011664* pair in *I. aquatica* belong to clade C24, which refers to anthocyanin biosynthesis. A comparison of interspecific collinearity among sweet potatoes with Arabidopsis and five other *Ipomoea* species was also analyzed to further explore the evolution of *R2R3-MYB* genes. There were 114, 175, 194, 171, 126, and 103 collinear blocks of *R2R3-MYB* genes containing 115, 242, 263, 245, 189, and 167 *IbR2R3-MYB* homologous gene pairs between *I. batatas* and *A. thaliana*, *I. trifida*, *I. triloba*, *I. nil*, *I. aquatica*, and *I. purpurea*, respectively (Figure 4 and Table S5). Interestingly, *I. batatas* vs. *I. trifida* and *I. batatas* vs. *I. triloba* gave a highly similar collinearity relationship (Figure 4b,c); and *I. batatas* vs. *I. nil,* and *I. batatas* vs. *I. purpurea* also exhibited a highly similar collinearity relationship (Figure 4d,f). However, the chromosomal correspondence between *I. batatas* and five other *Ipomoea* species and Arabidopsis was poor; this was mainly observed in different combinations of the chromosomes of one species with others. These results indicated that the *I. batatas* genome might have a larger chromosome level variation during the evolutionary process.

### 2.4. Gene Duplication Events and Ka/Ks Analysis of R2R3-MYB Genes in Six Ipomoea

**Figure 5:**
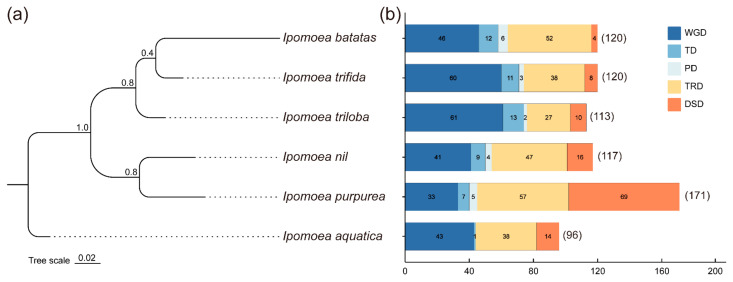
The number of *R2R3-MYB* gene pairs derived from different gene duplication events in the six *Ipomoea* species. (**a**) The phylogenetic relationship among the six *Ipomoea* species. The species tree was inferred from a concatenated alignment matrix of 9835 single-copy ortholog sequences across 6 *Ipomoea* species genomes. (**b**) The number of five models of duplicated gene pairs in each species. The gene duplication events include whole-genome duplication (WGD), tandem duplication (TD), proximal duplication (PD), transposed duplication (TRD), and dispersed duplication (DSD). The x-axis represents the number of duplicated gene pairs. The y-axis represents species.

To identify different modes of *R2R3-MYB* family gene duplication in six *Ipomoea* plants, five gene duplication events, including WGD, TD, PD, TRD, and DSD, were analyzed by running the DupGen_finder pipeline [55]. The results showed that a total of 737 *R2R3-MYB* gene duplication events were detected in these *Ipomoea* plant genomes (Figure 5 and Table S6), and the number of different gene duplication modes varied greatly. The WGD mode had the maximum number of duplication gene pairs with 284, followed by the TRD mode with 259 and the DSD mode with 121, indicating that the expansion of the *R2R3-MYB* gene family during evolution processes was mostly involved in WGD, TRD, and DSD events in *Ipomoea* species. In contrast, the TD mode, with 53, and the PD mode, with 20, were the minimum numbers of *R2R3-MYB* gene pairs. In addition, the number of WGDs and TRDs in each species was higher than TDs, PDs, and DSDs, except for *I. purpurea*. Furthermore, the distribution of five gene duplication events was largely similar in *I. batatas*, *I. trifida*, and *I. triloba*, and in *I. nil* and *I. purpurea*, which is consistent with the phylogenetic relationships of six *Ipomoea* species. These results indicated the importance of WGD and TRD modes for the *R2R3-MYB* gene family expansion in *Ipomoea* plants.

The non-synonymous substitution (Ka) to synonymous substitution (Ks) ratio (Ka/Ks) is an informative value of selection pressure during evolutionary history. Generally, Ka/Ks > 1 represents a positive selection, and Ka/Ks = 1 represents a neutral selection, while Ka/Ks < 1 represents a negative or purifying selection [48,55]. In this study, the Ka/Ks value of duplicated *R2R3-MYB* gene pairs was calculated and tested statistically, and all duplicated genes had a Ka/Ks ratio that was lower than 1 (Table S6), which indicated that these *R2R3-MYB* gene pairs experienced strong purifying evolutionary selection with limited functional divergence after duplication events. For the sweet potato *R2R3-MYB* gene family, the average Ka/Ks value of WGD, TD, PD, TRD, and DSD gene pairs were 024, 0.52, 0.46, 0.32, and 0.22, respectively. The TD gene pairs had the highest Ka/Ks value, suggesting that TD events had a higher evolution rate than other gene duplication events.

### 2.5. Phylogenetic Relationships, Gene Structures, and Conserved Motifs Analysis of 131 IbR2R3-MYB Genes

**Figure 6:**
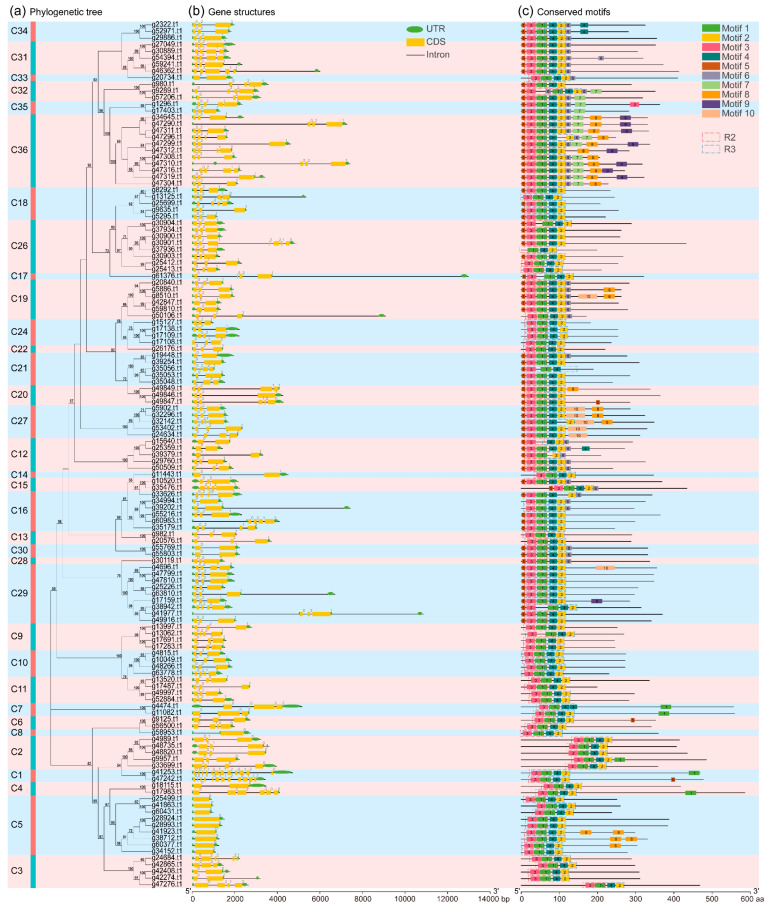
Phylogenetic relationships, exon-intron structures, and conserved motifs of the *R2R3-MYB* genes in sweet potatoes. (**a**) The phylogenetic tree of the 131 IbR2R3-MYB proteins. The tree was constructed by the maximum-like (ML) method of IQ-tree with 1000 bootstrap replicates. (**b**) The gene structure of the 131 *IbR2R3-MYB* genes. The yellow boxes, green ellipses, and black lines represent exons, untranslated regions (UTR), and introns, respectively. The bar scale at the bottom shows the length of the gene. (**c**) The conserved motifs of the 131 IbR2R3-MYB proteins. The boxes with different colors represent Motif 1–10, and the black solid line represents non-conserved regions. While the dashed boxes represent the R2 and R3 domains, their sequences logo shown in Figure S1. The bar scale at the bottom shows the length of the amino acids.

In this study, the phylogenetic tree, gene structure, and conserved motifs of 131 *IbR2R3-MYB* genes were combined to investigate sequence and structure characteristics. The results showed that the patterns of the gene exon-intron structures and protein amino acid motifs were strikingly similar among members within one clade but distinct between different clades (Figure 6). For example, the exon-intron structure analysis showed that the number of exons in *IbR2R3-MYB* genes varied from 1 to 12, with a mean of 3 (Figure 6b). Among them, 71 *IbR2R3-MYB* genes had 3 exons accounting for approximately 54%, 25 genes had 4 exons, 16 genes had 2 exons, 4 (*g46362.t1*, *g49847.t1*, *g33626.t1*, and *g24684.t1*) had 5 exons, 1 (*g17983.t1*) had 6 exons, 1 (*g30901.t1*) had 7 exons, and 2 (*g41253.t1* and *g47242.t1*) of clade C1 had a maximum number of exons with 12, while 9 genes from clade C5 only had 1 exon. In addition, the lengths of most *IbR2R3-MYB* genomic sequences were less than 4.0 kb with a mean of ~2.6 kb, and *g61376.t1* had the highest length of ~12.9 kb, while *g25499.t1* had only the lowest length of 923 bp. In brief, these results suggested that *IbR2R3-MYB* genes have evolutionary diversity between different phylogenetic clades and have evolutionary conservation within the same clade in terms of gene structure and length.

In this study, 10 conserved motifs, among 131 IbR2R3-MYB proteins, were further identified and named Motif 1 to Motif 10 (Figure 6c and Figure S2). R2R3-MYB proteins clustering together within the same clade generally have similar motif compositions (Figure 6c). For example, almost all IbR2R3-MYB proteins contained Motif 1, 2, 3, and 4, which form MYB DNA-binding domains. Motif 3 and Motif 1 formed the R2 domain, and Motif 4 and Motif 2 formed the R3 domain, which is located in the N-terminal of proteins. R2 and R3 domains were also identified and visualized (Figure 6c). Moreover, the number and arrangement of motifs varied in different clades, which is probably required for clade-specific functions. For instance, Motif 6 coupled with Motif 7 in C32 and C35 may be related to development, and Motif 8 alone in C5 or Motif 10 combined with Motif 8 in C27 may be related to abiotic stress responses. However, Motif groups 6/7/8/9 in C36 may suggest versatile biological functions. These results indicated the conservation and diversification of *IbR2R3-MYB’s* gene function.

### 2.6. Identification of R2R3-MYBs Related to Anthocyanin Biosynthesis in Sweet Potato

**Figure 7:**
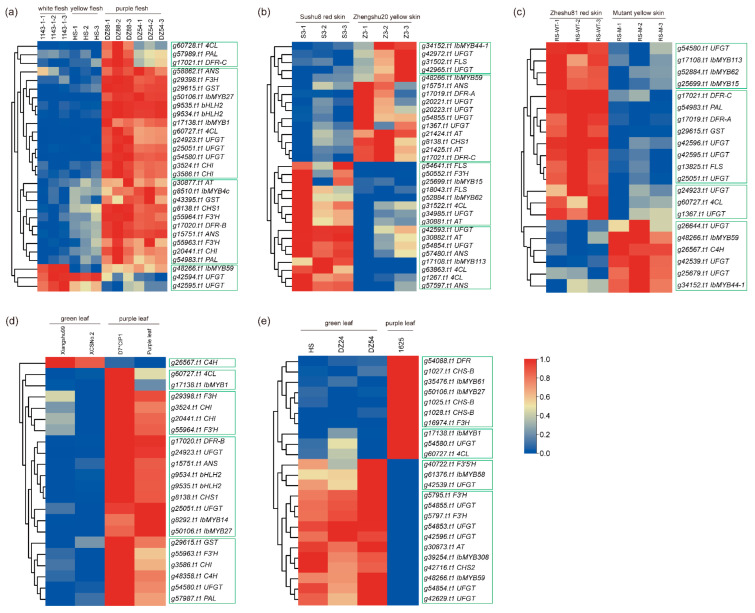
Expression patterns analysis of differentially expressed genes (DEGs) of *IbR2R3-MYB* and anthocyanin biosynthesis genes (ABGs) in different pigmented tissues of sweet potatoes. (**a**) *IbR2R3-MYBs* and ABGs in white, yellow, and purple fleshes. (**b**,**c**) *IbR2R3-MYBs* and ABGs in red and yellow skins. (**d**,**e**) *IbR2R3-MYBs* and ABGs in green and purple leaves. The Log_2_(FPKM + 1) values were row scaled and displayed according to the color code. The red and blue colors represent the highest and lowest expression levels, respectively. The hierarchical clustering and green boxes in each heatmap show similar expression patterns.

To investigate the key anthocyanin-related *IbR2R3-MYBs* in sweet potatoes, multiple public RNA-seq datasets representing different color variants in sweet potato leaf and tuberous root flesh and skin were analyzed [8,43,56,57]. These datasets focused on searching all potential candidate genes of anthocyanin biosynthesis in different sweet potato tissues. An integrated and comparative analysis was performed to identify the core anthocyanin-related *IbR2R3-MYBs* and anthocyanin biosynthesis genes (ABGs). The differentially expressed *IbR2R3-MYBs* and ABGs were determined via a Venn diagram analysis (Figure S3 and Table S7), and their relative expression profiles heatmaps were generated using TBtools software [58]. A total of 12 *IbR2R3-MYB* DEGs were identified and expressed in different color tissues of sweet potatoes (Figure 7). In purple tuberous root flesh, 4 *R2R3-MYBs*, 2 *bHLHs*, and 23 *ABGs* were identified, compared with white and yellow flesh (Figure 7a). The *g17138.t1* was identified as *IbMYB1* by BLASTP analyses, which belongs to the anthocyanin-activated C24(S6) clade. The *g50106.t1*(C19), *g8510.t1*(C19), and *g48266.t1*(C10) were identified as *IbMYB27*, *IbMYB4c*, and *IbMYB59*, respectively. *IbMYB1/g17138.t1*, *IbMYB27/g50206.t1*, and *IbMYB4c/g8510.t1* had similar expression patterns and showed a higher expression level in the purple flesh of ‘DZ88’ and ‘DZ54’, together with two *bHLH2* genes (*g9534.t1* and *g9535.t1*) and anthocyanin biosynthesis enzyme-coding genes, such as *4CL/g60727.t1*, *CHI/g3524.t1*, *F3H/g29398.t1*, *ANS/g58862.t1*, and *UFGT/g54580.t1*. In red tuberous root skin, three *IbR2R3-MYB* genes, *IbMYB113/g17108.t1*, *IbMYB62/g52884.t1*, and *IbMYB15/g25699.t1*, had relatively high or stronger expression levels in the red skin of ‘Sushu8’ and ‘Zheshu81’, while two genes (i.e., *IbMYB44-1/g34152.t1* and *IbMYB59/g48266.t1*) showed weak expression levels (Figure 7b, c). In purple leaf, *IbMYB1/g17138.t1*, *IbMYB27/g50106.t1*, and *IbMYB14/g8292.t1* showed a higher expression level in the purple leaf of ‘D7*CIP1’ and ‘Purple leaf’, which is comparable to the green leaf of ‘Xiangshu99’ and ‘XCSNo.2’ (Figure 7d). Coincidentally, *IbMYB1/g17138.t1*, *IbMYB27/g50106.t1*, and *IbMYB61/g35476.t1* were detected at a stronger expression level in the purple leaf of ‘1625’ while *IbMYB59/g48266.t1*, *IbMYB308/g39254.t1*, and *IbMYB58/g61376.t1* showed low expression levels (Figure 7e).

**Figure 8:**
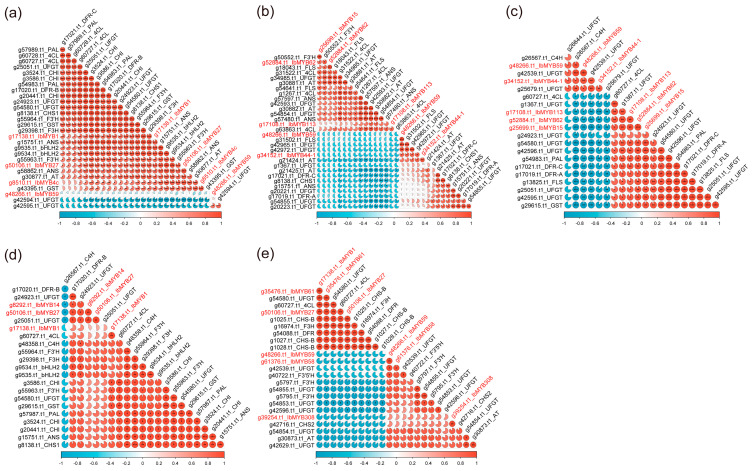
Pearson’s correlation analysis of differentially expressed genes (DEGs) of *IbR2R3-MYB* and anthocyanin biosynthesis genes (ABGs) in different pigmented tissues of sweet potatoes. (**a**) *IbR2R3-MYBs* and ABGs in white, yellow, and purple fleshes. (**b**,**c**) *IbR2R3-MYBs* and ABGs in red and yellow skins. (**d**,**e**) *IbR2R3-MYBs* and ABGs in green and purple leaves. The pie charts show the Pearson correlation coefficient values. The red and blue colors represent the positive and negative correlation, respectively. The asterisk shows the *p*-value, * *p* < 0.05, ** *p* < 0.01, and *** *p* < 0.001.

Furthermore, the expression levels of the above *IbR2R3-MYBs* and *ABGs* were used to perform a Pearson’s correlation analysis (Figure 8). The results showed that *IbMYB1/g17138.t1* and *IbMYB27/g50206.t1* were positively and significantly correlated to almost all ABGs, and they were negatively and significantly correlated to two *UFGTs* (*g42594.t1* and *g42595.t1*) in purple fleshes. Conversely, *IbMYB59/g48266.t1* had a significant positive correlation with *g42595.t1*, but it had no significant correlation with other ABGs (Figure 8a). A significant positive correlation between *IbMYB113/g17108.t1*, *IbMYB62/g52884.t1*, and *IbMYB15/g25699.t1* relative to 9 ABGs were observed in the red skin of ‘Zheshu81’, while *IbMYB59/g48266.t1* and *IbMYB44-1/g34152.t1* had significant negative correlations with 12 ABGs (Figure 8c). However, *IbMYB113/g17108.t1* also had a significant positive correlation with three ABGs (*g42593.t1*, *g30882.t1,* and *g63863.t1*) in the red skin of ‘Sushu8’ (Figure 8b). Similarly, *IbMYB1/g17138.t1* and *IbMYB27/g50206.t1* were positively and significantly correlated to at least five ABGs in purple leaves (Figure 8d,e). These results suggested that the identified *IbR2R3-MYBs* perform a core role in regulating the anthocyanin biosynthesis of sweet potatoes.

*IbMYB1/g17138.t1*, which was shown to be a transcriptional activator for enhancing anthocyanin accumulation in purple-fleshed sweet potatoes in many studies [8,47,48,49], was identified as a fundamental R2R3-MYB TF responsible for the anthocyanin biosynthesis of tuberous root flesh and leaves in sweet potatoes in this study. *IbMYB113/g17108.t1* had a relatively high expression level in red tuberous root skin but a very low or no expression in leaves and flesh, and it was positively and significantly correlated to most ABGs; thus, it was identified as a potential anthocyanin activator. *IbMYB62/g52884.t1*(C11) and *IbMYB15/g25699.t1*(C18) exhibited upregulated expression in pigmented tissues in sweet potatoes, suggesting their potential role in anthocyanin biosynthesis. However, the *IbMYB59/g48266.t1* was downregulated expression in pigmented leaves and tuberous root flesh and skin, but it had no significant correlations with ABGs, suggesting that it might not be involved in the anthocyanin biosynthesis pathway. In addition, *IbMYB27/g50206.t1*(C19), *IbMYB44-1/g34152.t1*(C5), and *IbMYB4c/g8510.t1*(C19) had been identified as R2R3-MYB repressor regulating the anthocyanin biosynthesis [51]. These results summarized that multiple *IbR2R3-MYBs* co-regulate anthocyanin biosynthesis in different sweet potato tissues, of which, *IbMYB1/g17138.t1* and *IbMYB113/g17108.t1* were anthocyanin-related core regulators.

## 3. Discussion

Sweet potato is an important food crop due to its strong adaptability, high yield potential, and high nutritional value [1]. The highly heterozygous hexaploid genome and the lack of knowledge of this genome limit the genetic studies of sweet potatoes [2]. A haplotype-resolved genome of the *I. batatas* cultivar ‘Taizhong 6’ was assembled [50] and used for molecular breeding. Two wild diploid species, *I.trifida* and *I. triloba,* had been reported as the closest relative of the hexaploid sweet potato, and they possessed a high-quality genome assembly [51]. In addition, three other *Ipomoea* species, *I. nil*, *I. purpurea*, and *I. aquatica,* were also sequenced [52,53,54]. These genome data contribute to the genetic study of sweet potatoes and genome evolution analysis in the *Ipomoea* species. In a previous study, some important enzyme-encoding genes (such as XTH, NBS, CDPK, SWEET, and SPL) [59,60,61,62,63] and TFs (such as GRAS, WRKY, bZIP, and MADS-box) [64,65,66,67,68] gene families were investigated in tuberous root development and the stress responses of *Ipomoea* species. However, a comprehensive and comparative molecular, evolutionary, and functional analysis of R2R3-MYB genes, which are indispensable for several developmental pathways and stress responses, is lacking in the *Ipomoea* species, even though the R2R3-MYB gene family has been identified in *I. aquatica* [69] and *I. nil* [70]. In the current study, six *Ipomoea* plants’ genomes information was employed in the identification and characterization of R2R3-MYB TFs, including protein physicochemical properties, phylogenetic relationships, chromosome localization, syntenic links, and gene duplication events. On this basis, the expression patterns of anthocyanin-related *IbR2R3-MYBs* in different colored sweet potato tissues were analyzed. The genome-wide comparative analysis of the R2R3-MYB TFs will facilitate the further study of their function and molecular breeding of sweet potatoes.

In this study, a total of 695 *R2R3-MYB* genes from the genomes of 6 *Ipomoea* plants were identified, and the number of identified *R2R3-MYBs* varied from 124 in *I. aquatica* to 133 in *I. trifida*, excluding 51 in *I. purpurea* (Table 1). There was no correlation between the *R2R3-MYB* genes and species phylogeny or the genome size that has been reported by previous studies [71]. Therefore, the difference may be due to the genome assembly strategy and quality of six *Ipomoea* species [2,72], and further improvements in these genome sequences are urgently needed. Furthermore, the identified R2R3-MYB proteins were clustered into 36 clades via an ML phylogenetic analysis (Figure 3), and the clade C25(S12) was Arabidopsis-specific, which was consistent with previous findings in five Solanaceae species. A total of 4 clades, including 102 R2R3-MYB proteins, which have no homologous protein in Arabidopsis, were identified as *Ipomoea*-specific clades, suggesting their species-specific role in *Ipomoea*, and their biological functions need to be further studied. Chromosomal localizations and distributions showed that the *R2R3-MYB* genes were unevenly distributed on all chromosomes in six *Ipomoea* species’ genomes, and some genes were located on unanchored scaffolds. An interspecific collinearity analysis of *R2R3-MYBs* showed that a total of 1223 collinearity gene pairs were detected among hexaploid *I. batatas* and 5 other diploid *Ipomoea* species, but their collinearity relationships were not as good as expected. Given their species’ phylogenetic relationships (Figure 5a), one reasonable explanation is that the hexaploid *I. batatas* genome might have undergone a larger chromosome rearrangement during the evolutionary process [60].

Gene and genome duplication processes are central to the species’ evolution and gene family expansion [55]. The present study showed that TRD was predominant in *I. batatas* and *I. nil*, and WGD was predominant in *I. trifida*, *I. triloba*, and *I. aquatica*, while DSD occurred in *I. purpurea*, suggesting that WGD, TRD, and DSD events play key roles in the expansion of *R2R3MYB* gene families in the *Ipomoea* species. Moreover, the Ka/Ks analysis showed that all duplicated *R2R3-MYB* gene pairs had a Ka/Ks ratio that was less than 1, which indicated that these genes experienced strong purifying selections in the *Ipomoea* species’ evolution process. Therefore, these results provided useful information for future research to understand the evolution of the *R2R3-MYB* gene family.

R2R3-MYBs are the major determinant regulators of the MBW(MYB-bHLH-WD40) complex, which fine-tune the spatial and temporal localization of anthocyanins by regulating the expression level of flavonoid biosynthesis pathway genes [17,73]. In *Arabidopsis thaliana*, four subgroups of R2R3-MYBs related to flavonoid syntheses have been identified, including S4, S5, S6, and S7 [9]. The S6 members contain the conserved KPRPR[S/T]F motif as anthocyanin activators to regulate anthocyanins biosynthesis at the transcription level, while the S4 members harbor repressive motifs, such as LIsrGIDPxT/SHRxI/L, pdLNLD/ELxiG/S, EAR, SID, and TLLLFR motifs, in C-terminals and can directly repress the flavonoid’s biosynthetic genes [74]. In this study, the phylogenetic analysis identified 13 IbR2R3-MYB members belonging to the C19(S4), C20(S7), and C24(S6) clades, and the FPKM values of g49847.t1(C20) and g15127.t1(C24) in all transcriptome data were less than 1 (Figure S4 and Table S8), indicating their nonfunction. Furthermore, R2R3-MYBs and the anthocyanin-related enzyme-encoding DEGs of colorful and colorless tissues in sweet potatoes were analyzed. In tuberous root flesh, *IbMYB1/g17138.t1* and *IbbHLH2* (*g9534.t1* and *g9535.t1*) form the transcriptional complex to activate downstream anthocyanin-related genes expression, such as *4CL/g60727.t1*, *CHI/g3524.t1*, *F3H/g29398.t1*, *ANS/g58862.t1*, *UFGT/g54580.t1*, and *GST/g29615.t1* (Figure 7a and Figure 8a), thus promoting the accumulation of anthocyanin. Meanwhile, *IbMYB1/g17138.t1* also performs an essential role in the anthocyanin biosynthesis of sweet potato purple leaves (Figure 7d,e and Figure 8d,e). In previous comparative transcriptome analyses of the red skin of ‘Sushu8’ [56] and ‘Zheshu81’ [8], *g17108.t1* had not been identified as anthocyanin-associated *R2R3-MYB* TF. A genome-wide comparative analysis of the *R2R3-MYB* gene family identified the *IbMYB113/g17108.t1* as an *R2R3-MYB* in this study. *IbMYB113/g17108.t1* may function in the activation of *4CL*(*g63863.t1* and *g60727.t1*), *DFR-A/g17019.t1*, *DFR-C/g17021.t1*, *UFGT*(*g42593.t1* and *g54584.t1*), and *AT/g30882.t1* (Figure 7b,c and Figure 8b,c), thus specifically regulating root skin coloration. Interestingly, multiple R2R3-MYB repressors may carry out competitive inhibition to prevent the excessive accumulation of anthocyanins [43], such as *IbMYB27/g50106.t1*, *IbMYB4c/g8510.t1*, and *IbMYB44-1/g34152.t1*, which suggests widespread negative feedback regulation mechanisms in the sweet potato anthocyanin metabolism. Based on these findings, it is speculated that the regulatory genes of anthocyanins in tuberous root flesh, skin, and leaves may be different and that *IbMYB1/g17138.t1* and *IbMYB113/g17108.t1* may regulate the spatiotemporal expression levels of anthocyanin-associated genes, thus resulting in tissue-specific anthocyanin accumulation in sweet potatoes. However, further molecular and genetic identification remains necessary in order to verify these genes’ true functions.

## 4. Materials and Methods

### 4.1. Genome-Wide Identification of R2R3-MYB Genes in Six Ipomoea Species

The whole genome sequences and genome annotation files of *Ipomoea batatas, Ipomoea trifida,* and *Ipomoea triloba* were downloaded from the *Ipomoea* Genome Hub (http://sweetpotao.com/, accessed on 1 September 2022) [50] and Sweetpotato Genomics Resource (http://sweetpotato.plantbiology.msu.edu/, accessed on 1 September 2022) [51]. However, the genome information of *Ipomoea nil* was downloaded from the following website: http://viewer.shigen.info/asagao/, accessed on 1 September 2022 [52]. Additionally, the genome databases of *Ipomoea purpurea* and *Ipomoea aquatica* were downloaded from the CoGe platform (https://genomevolution.org/coge/GenomeInfo.pl?gid=58735, accessed on 1 September 2022) [53] and National Genomics Data Center (https://ngdc.cncb.ac.cn/search/?dbId=gwh&q=PRJCA002216&page=1, accessed on 1 September 2022) [54], respectively. The longest coding sequence (CDS) and corresponding amino acid sequence of each gene were obtained by removing any alternatively spliced sequences by using the TBtools software [58].

To accurately identify all R2R3-MYB family members the six *Ipomoea* species, three different screening strategies were combined. First, the sequences of 126 AtR2R3-MYB proteins retrieved from TAIR (https://www.arabidopsis.org/, accessed on 1 September 2022) were used as queries for the BLASTP (E-value < 1 × 10^−5^) search by using TBtools software [58]. Subsequently, the MYB-like DNA-binding domain’s (PF00249) Hidden Markov Model (HMM) profile obtained from the Pfam database (http://pfam.xfam.org/, accessed on 3 September 2022) was used to search candidate proteins with conserved MYB domains by using the Advanced HMMER Search program in TBtools software [58]. Finally, all putative protein sequences further confirmed the presence of the R2R3 domain in three databases, including SMART (http://smart.embl-heidelberg.de/, accessed on 3 September 2022), Pfam (http://pfam.xfam.org/, accessed on 3 September 2022), and NCBI CD-search (https://www.ncbi.nlm.nih.gov/Structure/cdd/wrpsb.cgi, accessed on 3 September 2022). It should be noted that the protein sequences that did not have a complete R2R3 domain and those exhibiting redundant sequences were manually removed or corrected. The physicochemical properties of all identified R2R3-MYB members, including protein length, molecular weight (MW), isoelectric point (pI), instability index, aliphatic index, and grand average of hydropathicity (GRAVY) were analyzed via the ProtParam tool of the Expasy website (https://web.expasy.org/protparam/, accessed on 14 September 2022), and their subcellular localization was also predicted by using the WoLF PSORT website (https://wolfpsort.hgc.jp/, accessed on 14 September 2022).

### 4.2. Multiple Sequences Alignment and Phylogenetic Evolution Analysis of R2R3-MYBs

All R2R3-MYB protein full-length sequences from six *Ipomoea* species and *Arabidopsis thaliana* containing the AtCDC5 outgroup protein were used to perform phylogenetic evolution analyses. Multiple sequence alignments were carried out using the MAFFT v7.505 program [75] with default parameters, and a maximum-likelihood (ML) phylogenetic tree was constructed using the IQ-TREE v1.6.12 program [76] with auto parameters and 1000 bootstraps. The 131 IbR2R3-MYB proteins also were used to generate phylogenetic trees by using the same methods. The phylogenetic trees were subsequently visualized using the ChiPlot online tool (https://www.chiplot.online/index.html, accessed on 27 February 2023).

### 4.3. Chromosome Distribution and Syntenic Analysis of R2R3-MYBs in Six Ipomoea Species

The physical position and chromosomal distribution information of six *Ipomoea* species *R2R3-MYB* genes were provided in the corresponding genome annotation files (GFF3). The syntenic relationships of *R2R3-MYB* genes in six *Ipomoea* intraspecies and interspecies were analyzed using the One Step MCScanX program in the TBtools software with default parameters [58]. Furthermore, the Advanced Circos function in TBtools software was used to visualize the distribution information and syntenic results of six *Ipomoea* species *R2R3-MYB* genes across all chromosomes. It is worth noting that the genes distributed on unanchored scaffolds were excluded.

### 4.4. Gene Duplication Events and Ka/Ks Analysis of R2R3-MYBs in Six Ipomoea Species

OrthoFinder v2.5.4 [77] constructed the taxonomic tree of the six *Ipomoea* species. The gene duplication events of six *Ipomoea* species *R2R3-MYB* duplicated gene pairs derived from whole-genome duplication (WGD), tandem duplication (TD), proximal duplication (PD), transposed duplication (TRD), and dispersed duplication (DSD) were detected by running the DupGen_finder pipeline, in which *Arabidopsis thaliana* was selected as the outgroup in order to identify duplicated gene pairs. In addition, the nonsynonymous (Ka) and synonymous substitution rates (Ks) of syntenic gene pairs were calculated using Tbtools with the Nei–Gojobori (NG) method [58].

### 4.5. Gene Structure and Conserved Protein Motif Analysis of R2R3-MYBs in Sweet potato

The GFF3 annotation file of the *I. batatas* genome containing information about the gene structure was used to identify the exons and introns of 131 *IbR2R3-MYB* genes. The MEME online tools (https://meme-suite.org/meme/, accessed on 28 September 2022) were used to identify the conserved motifs of IbR2R3-MYB proteins, with a maximum number of motifs to 10 and other default parameters. The gene structure and conserved motifs were subsequently visualized by TBtools software [58].

### 4.6. Identification of Anthocyanin-Related R2R3-MYBs Via RNA-Seq Data in Sweet potato

Multiple public RNA-seq datasets were downloaded from the sequence read archive (SRA) of NCBI, and these datasets were employed to explore the expression patterns of anthocyanin-related *IbR2R3-MYB* genes in sweet potato different tissues. Of which, projects PRJNA562409 and PRJNA772025 comprised the transcriptome analysis of coloration mutation of root skin in sweet potato cultivar ‘Sushu8’ [56] and ‘Zheshu81’ [8], respectively. PRJNA721067 was a transcriptome analysis that included many sweet potato cultivars, and they present a typical color variant of purple leaves in the aerial part or purplish rings/spots in the storage root flesh [7]. In addition, our previous transcriptome study of four sweet potato cultivars with different pigmentation phenotypes in root flesh (PRJNA881010, PRJNA881014, PRJNA881013, and PRJNA881012) [57] was also used to identify the expression levels of *IbR2R3-MYB* genes.

The Hisat2 software [78] was used to align clean reads to the sweet potato genome. The fragments per kilobase million (FPKM) was measured to estimate *IbR2R3-MYB* gene expression levels by using the StringTie program [78]. Differentially expressed genes (DEGs) were identified by using the DESeq2 R package with an adjusted *p* value < 0.01 and absolute log2-fold change > 1 as the criteria [78]. First, the differentially expressed genes in each comparation and 131 identified *IbR2R3-MYBs* were selected to identify the candidate *IbR2R3-MYBs* and anthocyanin biosynthesis genes (ABGs) via a Venn diagram analysis. Next, the heatmaps of the expression levels of candidate *IbR2R3-MYBs* and anthocyanin biosynthesis genes (ABGs) were visualized in different colored sweet potato tissues using the TBtools software [58]. Furthermore, Pearson’s correlation analyses were also performed and visualized using the corrplot R package.

## 5. Conclusions

In summary, this study presented a comprehensive genome-wide analysis of the *R2R3-MYB* gene family in six *Ipomoea* plants. A total of 695 typical *R2R3-MYB* genes were identified, including 131 *IbR2R3-MYB* genes of the sweet potato. An ML phylogenetic analysis divided these genes into 36 clades, and 4 clades, including 102 members, were identified as *Ipomoea*-specific clades. WGD, TRD, and DSD events were the primary forces driving the R2R3-MYB gene family expansion of *Ipomoea* plants, and these duplicated genes experienced strong purifying selections. Furthermore, based on multiple RNA-seq data, two core *IbR2R3-MYB* genes (*IbMYB1/g17138.t1* and *IbMYB113/g17108.t1*) were identified as responsible for regulating tissue-specific anthocyanin accumulation in sweet potato. These results can enhance our understanding of the *R2R3-MYB* gene family of sweet potato and provide novel *R2R3-MYB* gene information for anthocyanin metabolic engineering.

## Figures and Tables

**Table 1:** Genomic information and identified R2R3-MYB gene numbers in six *Ipomoea* species.

Scientific Name	Common Name	Ploidy	Chromosome Number	Genome Size	Genome Gene Number	R2r3-Myb Genes Number
*I. batatas*	Sweet potato	hexaploid	15	451 Mb	64,295	131
*I. trifida*		diploid	15	477 Mb	32,301	133
*I. triloba*	Trilobed morning glory	diploid	15	447 Mb	31,426	129
*I. nil*	Japanese morning glory	diploid	15	707 Mb	42,783	127
*I. purpurea*	Common morning glory	diploid	15	581 Mb	53,979	51
*I. aquatica*	Water spinach	diploid	15	494 Mb	29,606	124

Note: Genome size and gene number are estimated based on the final assembly version used in this study.

**Table 2:** The R2R3-MYB members number of each clade in each species and their major functions of each clade.

Clade	Classification in Arabidopsis	Function Annotation	*At*	*Ib*	*Itf*	*Itb*	*In*	*Ia*	*Ip*	Total
C1	S28	development/stomatal formation	2	2	2	2	2	2	2	14
C2	S25	development/female gametogenesis	7	5	5	3	8	6	11	45
C3	S21	development/specialized metabolism/cell wall thickening	8	5	6	5	5	5	4	38
C4	S23	abiotic stress/salt stress	3	2	2	2	2	2	1	14
C5	S22	abiotic stress/drought, salt, and cold stress	4	9	8	8	8	8	10	55
C6	S26	development/sperm cell formation/pollen formation	1	2	2	2	2	2	2	13
C7	S18 and S77	development/stamen development	7	2	4	4	4	3	3	27
C8	S27	development/leaf patterning	1	1	1	1	1	1	1	7
C9	S17	abiotic stress	3	4	3	3	3	3	1	20
C10	S78	abiotic stress/potassium stress	3	4	1	2	1	1	2	14
C11	S19 and S20	development/abiotic stress	9	4	5	5	6	5	4	38
C12	S8	secondary wall/lignin biosynthesis/abiotic stress	6	5	6	6	6	6	0	35
C13	S16	development/hypocotyl elongation	3	2	2	2	2	2	0	13
C14	S32	phenylpropanoid-derived secondary metabolites/secondary cell wall biosynthesis	2	1	1	2	1	2	1	10
C15	S13	lignin/triterpenoid/cellulose biosynthesis	4	2	3	3	3	3	0	18
C16	S30 and S31	starch biosynthesis/fructan synthesis/secondary cell wall biosynthesis	3	6	8	8	7	8	0	40
C17	S3	phenylpropanoid-derived secondary metabolites/flavonoid/lignin biosynthesis	4	1	2	1	1	1	0	10
C18	S2	abiotic stress	3	5	5	5	4	5	1	28
C19	S4	phenylpropanoid-derived secondary metabolites/proanthocyanin/lignin biosynthesis	6	6	6	7	6	7	2	40
C20	S7	phenylpropanoid-derived secondary metabolites/flavonol biosynthesis	3	3	3	2	2	3	0	16
C21	NO	function unknown	0	5	4	4	4	4	0	21
C22	S15	development/root hair development	4	1	1	1	1	1	0	9
C23	S44	phenylpropanoid-derived secondary metabolites/proanthocyanin biosynthesis	1	0	1	1	3	2	3	11
C24	S6	phenylpropanoid-derived secondary metabolites/anthocyanin biosynthesis	4	4	6	5	6	5	0	30
C25	S12	glucosinolate biosynthesis	6	0	0	0	0	0	0	6
C26	NO	function unknown	0	8	9	8	6	5	2	38
C27	S1	abiotic stress/specialized metabolism	5	5	4	4	4	4	0	26
C28	S33 and S46	development/anther/pollen development	2	1	2	1	2	2	0	10
C29	S14	abiotic stress/specialized metabolism/development	6	9	11	10	9	12	0	57
C30	NO	function unknown	0	2	2	2	2	2	0	10
C31	S11	abiotic stress	4	5	3	3	4	4	0	23
C32	S36	development/inflorescence development/seed germination	1	3	3	3	3	3	0	16
C33	S10	suberin biosynthesis	2	1	1	1	1	1	1	8
C34	S24	development/lateral root development	3	3	1	2	2	2	0	13
C35	S9	development/trichome branching/cuticle formation	2	2	2	2	1	2	0	11
C36	NO	function unknown	0	11	8	9	5	0	0	33

Note: The classification and function annotation of R2R3-MYB members were obtained from references [9,14].

## Data Availability

Not applicable.

## Supplementary Materials

The following supporting information can be downloaded at: https://www.mdpi.com/article/10.3390/plants12081731/s1. Figure S1. The sequence logo of R2 (a) and R3 (b) MYB domain; Figure S2. The sequence logos of conserved motifs (Motif 1-10) of the 131 IbR2R3-MYB proteins; Figure S3. The identification of anthocyanin-related *IbR2R3-MYB* DEGs in sweetpotato pigmented tissues via Venn diagram analysis; Figure S4. A heatmap of expression levels of 13 *IbR2R3-MYB* genes belong to C19(S4), C20(S7), and C24(S6) clades in sweetpotato pigmented tissues; Table S1. Physicochemical properties and subcellular localization of all identified R2R3-MYB proteins in six *Ipomoea* species; Table S2. Sequences and clades of all identified R2R3-MYB proteins in six *Ipomoea* species and Arabidopsis; Table S3. Gene feature of all identified *R2R3-MYB* genes in six *Ipomoea* species; Table S4. Syntenic *R2R3-MYB* gene pairs among each *Ipomoea* species; Table S5. Collinear *R2R3-MYB* gene pairs between sweetpotato and Arabidopsis and other five *Ipomoea* species; Table S6. *R2R3-MYB* duplicated gene pairs and Ka/Ks value of six Ipomoea species; Table S7. FPKM value of *IbR2R3-MYB* and *ABG* DEGs in purple tuberous root flesh, red skin, and purple leaf; Table S8. FPKM value of 13 *IbR2R3-MYB* genes belong to C19(S4), C20(S7), and C24(S6) clades.
