# Supplementary material for: Genome-Wide Comparative Analysis of the R2R3-MYB Gene Family in Six Ipomoea Species and the Identification of Anthocyanin-Related Members in Sweet Potatoes
Source: Plants (Basel). 2023 Apr 21;12(8):1731. doi: 10.3390/plants12081731 (PMC10140993; doi:10.3390/plants12081731)
Supplement: Supplementary file 1 [file plants-12-01731-s001.zip › supplementary figure s1-4.pdf]

(a) R2 domain

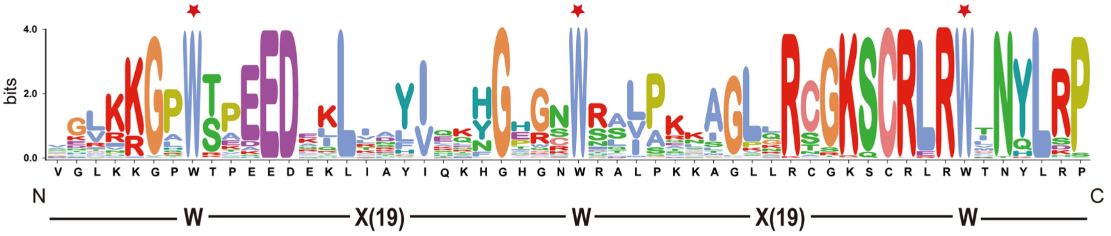

(b) R3 domain

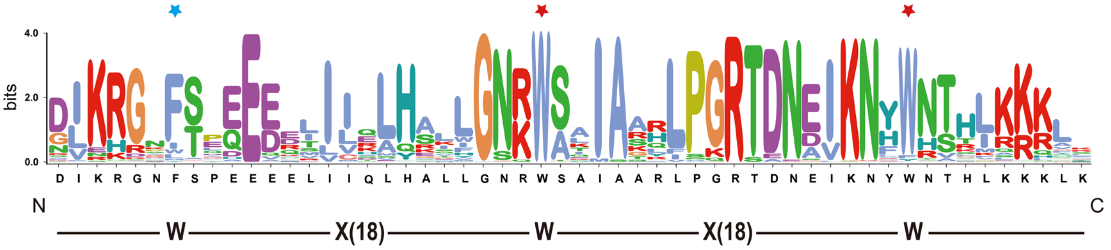

Figure S1. The sequence logo of R2 (a) and R3 (b) MYB domain. The bit score exhibits the information content for each position in the sequence. The MYB domains are based on full-length alignments of 131 IbR2R3-MYB proteins. The red stars show the highly conserved tryptophan (W) residues, while the bule star show the relatively conserved phenylalanine (F) residues to replace W residue in R3 domain.

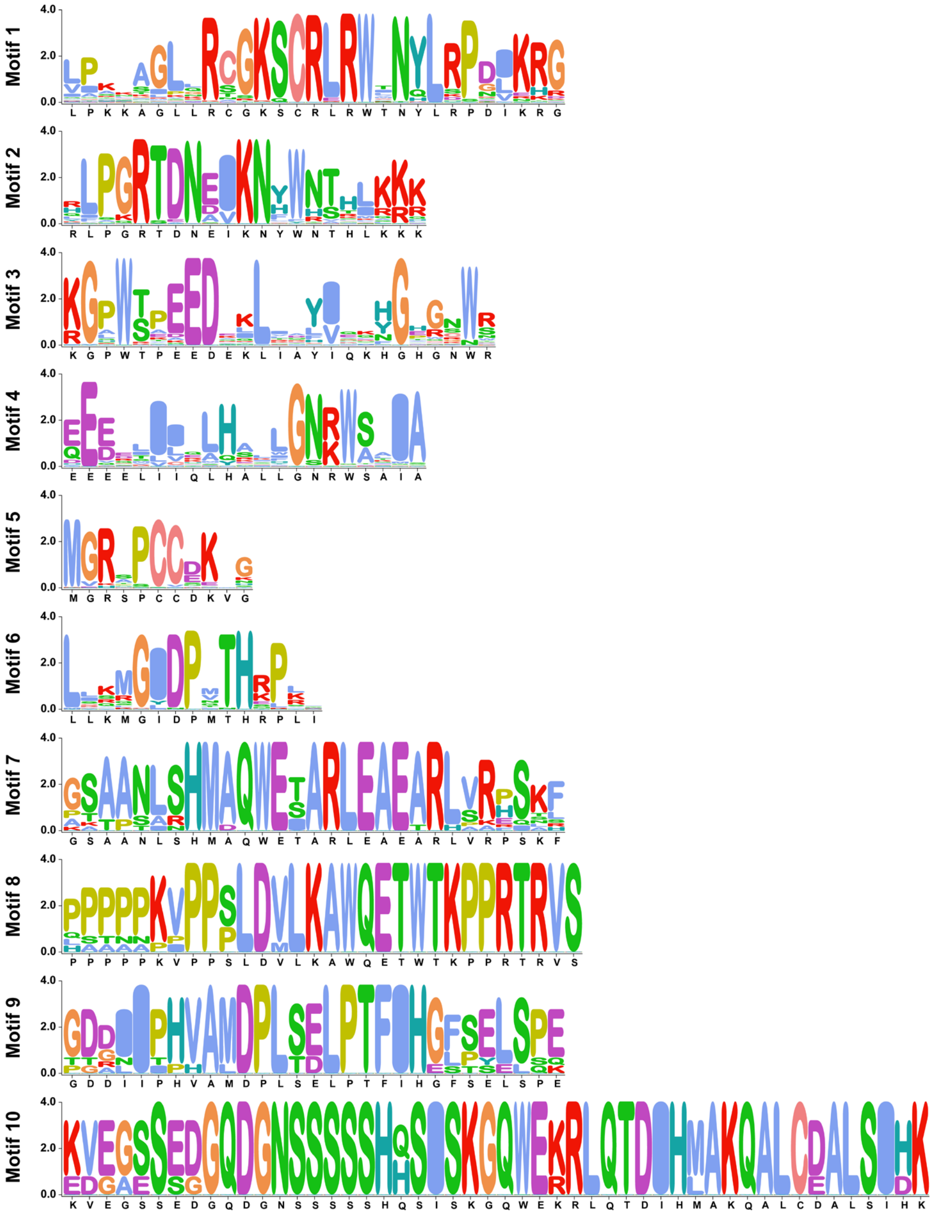

Figure S2. The sequence logos of conserved motifs (Motif 1-10) of the 131 IbR2R3-MYB proteins. The y-axis bit score exhibits the information content for each position in the sequence. The ten motifs were identified via MEME online tools, and the sequence logos were visualized via TBtools software.

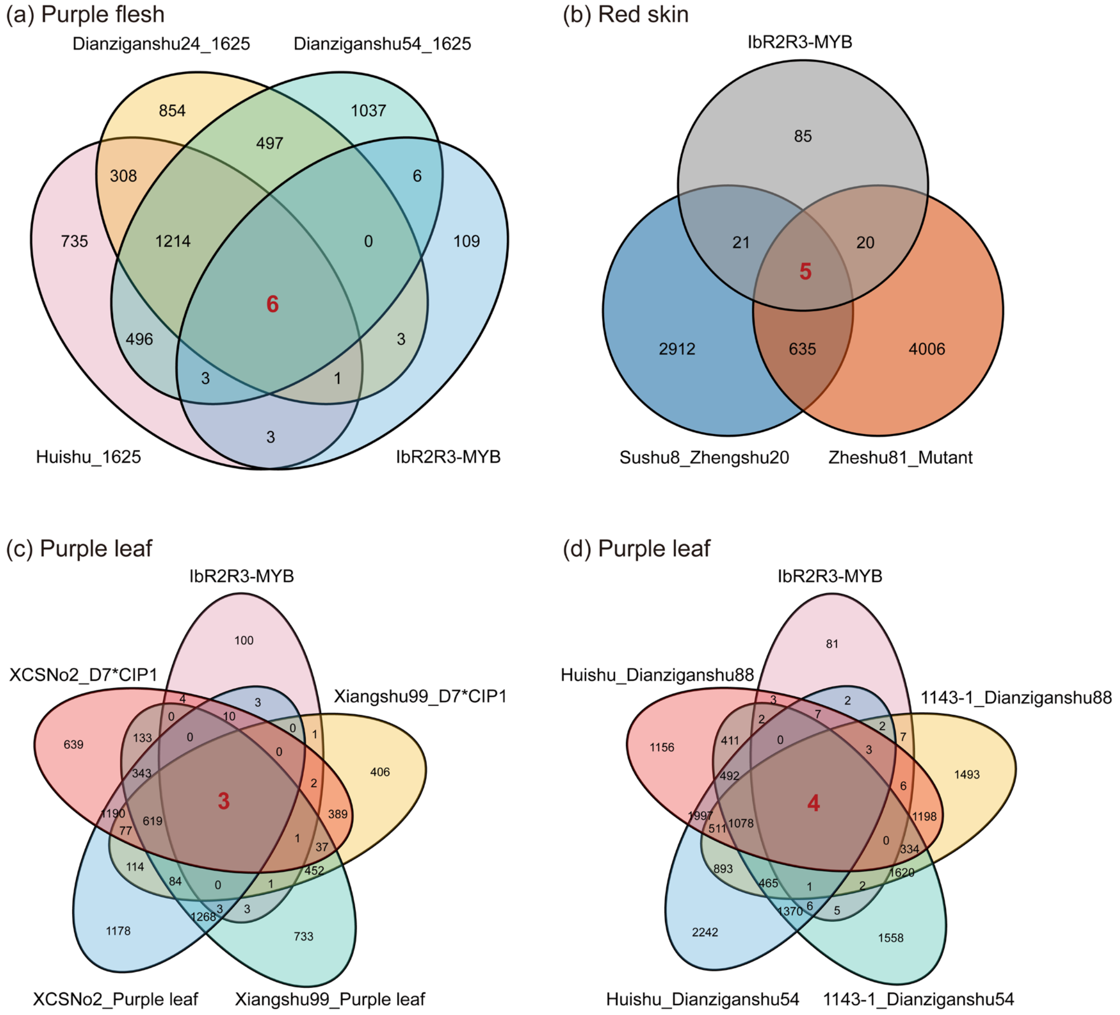

Figure S3. The identification of anthocyanin-related *IbR2R3-MYB* DEGs in sweetpotato pigmented tissues via Venn diagram analysis. (a) *IbR2R3-MYB* DEGs in purple flesh. (b) *IbR2R3-MYB* DEGs in red skin. (c-d) *IbR2R3-MYB* DEGs in purple leaf.

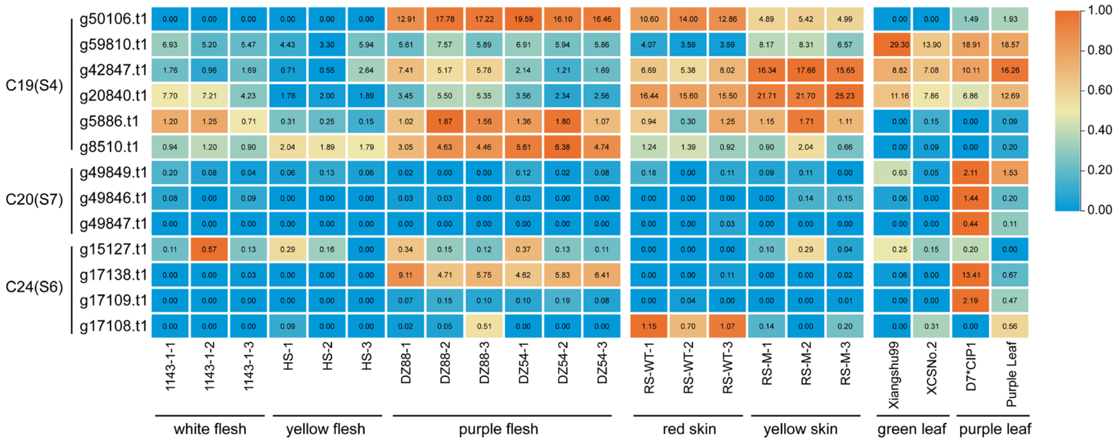

Figure S4. A heatmap of expression levels of 13 IbR2R3-MYB genes belong to C19(S4), C20(S7), and C24(S6) clades in sweetpotato pigmented tissues. The Log_2(FPKM+1) values were row scaled and displayed according to the color code. The orange and cyan colors represent the highest and lowest expression levels, respectively. The FPKM original values were showed on corresponding rectangles.
